# Cognitive performance in older elderly men with late-life depression and cardiovascular comorbidities: symptomatological correlation

**DOI:** 10.1186/1744-859X-12-36

**Published:** 2013-11-15

**Authors:** Yun-Hsuan Chang, Mu-En Liu, Chih-Chun Huang, Yan-Chiou Ku, Sheng-Yu Lee, Shiou-Lan Chen, Wen-Chien Liu, Ru-Band Lu

**Affiliations:** 1 Division of Clinical Psychology, Institute of Allied Health Sciences, National Cheng Kung University, Tainan 704, Taiwan; 2 Department of Psychiatry, College of Medicine, National Cheng Kung University, Tainan 704, Taiwan; 3 Department of Psychiatry, National Cheng Kung University Hospital, Tainan 704, Taiwan; 4 Institute of Behavioral Medicine, College of Medicine, National Cheng Kung University, Tainan 704, Taiwan; 5 Department of Psychiatry, Taipei Veteran General Hospital, Taipei 11217, Taiwan; 6 Department of Nursing, Kaohsiung Veterans General Hospital, Kaohsiung 813, Taiwan; 7 Department of Psychiatry, Yuli Veterans Hospital, Hualien 970, Taiwan; 8 Addiction Research Center, National Cheng Kung University, Tainan 704, Taiwan

**Keywords:** Cardiovascular comorbidity, Cognitive function, Late-life depression, Major depression, Older elderly

## Abstract

**Background:**

Whether depression or cardiovascular disease would have a greater effect on worsening cognitive impairment in the burgeoning older elderly population is uncertain. Which disorder causes greater cognitive impairment was investigated.

**Methods:**

A cross section of 207 cognitively impaired older elderly (≥75 years old) men was recruited from outpatient clinics in southern Taiwan between 2004 and 2008. Their medical charts were reviewed for their history of medical illnesses, and those undergoing a current major depressive episode were screened using the Mini-International Neuropsychiatric Interview. Four groups of men were enrolled: 33 healthy controls (HC), 101 cognitively impaired patients with cardiovascular comorbidities (CVCs), 34 patients with late-life depression (LLD), and 49 patients with LLD and cardiovascular comorbidities (LLD + CVC). Several neuropsychological tests (e.g., Mini-Mental State Examination (MMSE), WCST, and Trail Making Test (TMT) parts A and B) were used to assess the participants.

**Results:**

Cognitive function scores were highest in the HC group and lowest in the LLD + CVC group. There were no significant differences between the two groups with LLD comorbidity, and LLD was mostly associated with cognitive performance. LLD + CVC group members had the lowest recall memory, but their overall MMSE score was not significantly different. Moreover, this group had a higher but nonsignificantly different perseverative error than did the LLD group. Similarly, the LLD + CVC group was nonsignificantly slower at the TMT-A and TMT-B tasks than was the LLD group.

**Conclusions:**

LLD worsens neuropsychological function more than cardiovascular comorbidities do.

## Background

As the aging population dramatically increases, the topic of aging has attracted attention for various reasons. The percentage of elderly people (≥65 years old) in Taiwan was around 10.63 % of the total population (23 million) in Taiwan in 2010 [1] because of a significant increase in the number of older elderly (≥75 years old). The more medical disabilities the older elderly have, the more vulnerable they are to depression and the more social support they require than do younger elderly people (60–74 years old) [2]. The symptoms they present with sometimes indicate complex and variable psychiatric problems. Because current knowledge about geriatric illness is limited, it is still challenging for physicians to identify comorbid medical and psychiatric illnesses early in elderly patients.

Depression is the leading cause of geriatric psychiatric problems. It increased with age in a population of oldest elderly (≥80 years old) Han Chinese in Hong Kong [3]. The high prevalence of major depression seems to be a significant risk factor for suicide in the older elderly [4]. According to epidemiological data, some medical illnesses are also associated with depression, such as cardiovascular comorbidities (CVCs) [5]. One imaging study reports that specific cerebrovascular lesions are related to late-life depression (LLD) [6]. Moreover, the management and prognosis of underlying medical illnesses can be affected by major depression.

Both depression and cardiovascular diseases (CVDs) are risk factors for cognitive impairment [7], but the various neuropsychological mechanisms underlying LLD can lead to different patterns of cognitive impairment [8]. The current association between depression and cognitive impairment agrees with previous studies [9,10]. Cardiovascular disease risk factors, such as hypertension and diabetes, are associated with poorer neuropsychological functioning as well as with increased rates of cognitive decline [11]. In addition, the depressed elderly with executive dysfunction are more likely to have a cardiovascular comorbidity [12]. Both LLD and CVD seem to cause a decline in cognitive function; however, which has the greater effect is not known.

Therefore, we investigated whether LLD or CVCs (not all of our participants with CVCs had full-blown cardiovascular diseases) have greater effect on cognitive impairment. The Chinese version of the Geriatric Depression Scale, a useful tool for assessing depression in Chinese older elderly who live alone [13], was used to explore this issue.

## Methods

### Participants and procedures

This study was approved by the Institutional Review Board of National Cheng Kung University Hospital. Participants were recruited from among newly admitted residents of Taiwan National Veterans' Care Homes in Tainan and Kaohsiung. They had voluntarily applied for residency in Veterans' Care Homes. The majority were ambulatory, physically capable, and able to provide for their own daily needs with no assistance from others. Only a few were physically disabled and required a wheelchair, cane, or other ambulatory equipment, or needed other assistance.

Two hundred seventeen new residents, who had completed health examinations between August 2004 and February 2008 at clinics affiliated with Veterans' Care Homes, volunteered for this study. The study procedures were fully explained, and written informed consent was obtained from all.

The inclusion criteria were as follows: (1) 75 years old or older, (2) capable of verbal communication, and (3) able to speak Mandarin or Taiwanese. Participants diagnosed with major depressive disorder or with a first depressive episode were assigned to one of the two LLD groups. Participants with one or more CVCs, *viz.*, hypertension, diabetes, coronary heart disease, cigarette smoking, atrial fibrillation, and left ventricular hypertrophy, were assigned to the CVC group. For this group, medical measures were completed by a physician based on interviews and medical chart review. Participants diagnosed with both LLD and CVCs were assigned to the LLD + CVC group. The exclusion criteria were factors that might interfere with the presentation of depressive symptoms: (1) a history of dementia or suspected dementia, (2) a current diagnosis of psychiatric disorders other than major depressive disorder, (3) current use of antipsychotics or mood stabilizers, or (4) current substance abuse. Thirty-three healthy participants were also recruited as controls (HCs).

All volunteers were interviewed by an attending psychiatrist for an initial evaluation and then by psychiatrists and psychologists using the Mini-Mental State Examination (MMSE), the Geriatric Depression Scale (GDS), and the Mini-International Neuropsychiatric Interview (MINI). All patients in the two LLD groups were given an antidepressant and a small dose of benzodiazepines. To increase inter-rater reliability, all the interviewers were intensively trained on how to use the interview instruments, and all were closely supervised by one of the authors (RBL). All demographic data, medical charts, and histories of medical illnesses were reviewed, and then the results of the health examinations were analyzed.

### Measurements

#### MMSE

The Chinese version of the MMSE with a standardized norm was used to screen the volunteers. Those suspected of having dementia, i.e., those with scores lower than the two literacy-related cutoff scores—23–24 for the literate and 13–14 for the illiterate (maximum possible score, 30) [14]—were excluded.

### Diagnosis and severity of depression

The Chinese version of the MINI was used to evaluate current depressive episodes and other psychiatric diagnoses [15]. The diagnostic criteria of the MINI are based on the *Diagnostic and Statistical Manual of Mental Disorders*, *Fourth Edition* (DSM-IV). The revised Chinese 15-item GDS was used to measure the severity of depression. The Chinese versions of the MINI and the GDS have been validated [16].

### Neuropsychological function

#### Wisconsin card sorting test

The Wisconsin Card Sorting Test (WCST) measures the ability to perform certain types of executive functions: categorization, abstract reasoning, set creation, set switching, strategic planning, and modulation of impulsive responding [17]. Participants are required to try out different rules to determine the correct method of sorting the cards into piles in front of four stimulus cards. The participants were instructed to infer the matching principle from the feedback provided, 'correct’ or 'incorrect’, depending on whether they guessed the rule correctly or not. The cards could be matched by number (1, 2, 3, or 4), color (yellow, green, blue, or red), or shape of the symbols (star, triangle, circle, or cross). The rule is applied for a run of trials and then changed without warning [18]. The inter-rater reliability is 0.88–0.93, within-rater reliability is 0.91–0.96, and test-retest reliability is 0.57. Performance on the WCST was scored in terms of the total number of errors (TNE), perseverative errors (PE), conceptual level response (CLR), number of categories completed (NCC), and trials to complete the first category (TCC).

#### Trail Making Test

The Trail Making Test (TMT) [19] is a visuomotor task that consists of two parts: in part A, the participant is asked to draw lines to connect consecutively numbered circles on one worksheet, and then in part B, to connect the same number of consecutively numbered and lettered circles by alternating between the two sets, which involves frequent switching between two mental sets. The instruction for both parts was to perform the test as correctly and as quickly as possible, and the scoring was based on completion time [19].

Because visual search and general psychomotor performance are relevant for both parts of the test, calculating the difference in scores between parts A and B minimizes these interfering effects. The TMTB-A score is well established as an instrument for assessing executive function with special emphasis on set shifting, while some researchers use the TMTB/A ratio for the same purpose [20]. A large-scale (*n* = 285) comparative assessment of the two scores yielded better discriminatory power of the TMTB-A score with respect to the detection of performance differences between the young and the elderly [21].

### Statistical analyses

Group differences in categorical and numerical variables were analyzed using a *χ*^2^ test and the Kruskal-Wallis test or analysis of variation (ANOVA). In addition, Fisher's least significant difference test and the Mann–Whitney *U* test were used for the *post hoc* comparisons. The two-tailed significance level was set at 0.01 to reduce type I errors of multiple comparisons. All the analyses were done using SPSS 18.0 (SPSS Institute, Chicago, IL, USA).

## Results

### Demographic data

All participants in the study were male. Their mean age was 81.23 ± 4.20 years, mean education level was 5.05 ± 4.65 years, and mean MMSE score was 24.43 ± 2.82. There were no significant differences in age (*F* = 0.83, *p* = 0.48), education levels (*F* = 2.15, *p* = 0.10), and MMSE scores (*F* = 0.86, *p* = 0.46) between groups. The LLD and LLD + CVC groups had significantly (*p* < 0.0005) higher GDS scores than did the HC and CVC groups (Table 1).

**Table 1:** Demographic data

	**Groups**				**Statistics**	
	**HC (*n*** **= 33)**	**CVC (*n*** **= 101)**	**LLD (*n*** **= 34)**	**LLD + CVC (*n*** **= 49)**	***F *** **(*p *** **value)**	***Post hoc***
Age (years)	81.41 ± 3.89	81.02 ± 4.22	80.53 ± 3.28	81.80 ± 4.67	0.83 (0.48)	**-**
Education (years)	5.23 ± 4.01	5.93 ± 5.14	4.79 ± 3.98	3.53 ± 3.98	2.15 (0.10)	-
MMSE score	24.73 ± 3.03	24.78 ± 2.55	24.35 ± 2.57	23.72 ± 3.16	0.86 (0.46)	-
GDS	2.25 ± 1.87	2.44 ± 1.31	7.47 ± 1.81	6.83 ± 2.60	143.08 (<0.0005)	LLD, LLD + CVC > HC, CVC (*p* < 0.0005)

Data are presented as mean ± standard deviation (SD). HC, older elderly healthy controls; CVC, older elderly men with cardiovascular comorbidities; LLD, older elderly men with late-life depression; LLD + CVC, older elderly men with late-life depression and cardiovascular comorbidities; GDS, Geriatric Depression Scale.

### Neuropsychological data

Although these participants were not suspected of having dementia, the MMSE subscales were evaluated to investigate which part of the cognitive function might be a more sensitive index in these older elderly. There was a significant difference in recall memory but not in the other indices (Table 2). Further, the proportion of participants that completed all three recall objects was compared; no participants in the LLD and LLD + CVC groups received a full score on that item, although the differences were not significant (*χ*^2^ = 5.09, *p* = 0.17). Although education can be a factor in the performance on the WCST [22], a multiple analysis of variance (MANOVA) showed no significant differences in education level between the four groups in this study.

**Table 2:** Comparison of MMSE subscale among four groups of older elderly men

	**HC (*n*** **= 33)**	**CVD (*n*** **= 101)**	**LLD (*n*** **= 34)**	**LLD + CVC (*n*** **= 49)**	**ANOVA ( *F *, *p *** **value)**	***Post hoc***^**a**^
Orientation	7.73 ± 1.13	7.84 ± 1.08	7.82 ± 0.87	7.92 ± 1.10	(*F* = 0.22, *p* = 0.89)	-
Registration	2.48 ± 0.57	2.59 ± 0.51	2.47 ± 0.56	2.45 ± 0.54	(*F* = 1.07, *p* = 0.36)	-
Attention	3.94 ± 0.61	3.90 ± 0.61	3.91 ± 0.57	3.71 ± 0.71	(*F* = 1.27, *p* = 0.28)	-
Recall memory	2.09 ± 0.38	1.98 ± 0.55	1.94 ± 0.34	1.61 ± 0.64	(*F* = 7.26, *p* < 0.0005)	HC > LLD + CVC, CVC > LLD + CVC
Language	7.06 ± 0.79	6.97 ± 0.69	7.24 ± 0.74	7.02 ± 0.78	(*F* = 1.13, *p* = 0.34)	-
Spatial concept	0.88 ± 0.33	0.94 ± 0.24	0.91 ± 0.29	0.92 ± 0.28	(*F* = 0.46, *p* = 0.71)	-

Data are presented as means ± SD. HC, healthy controls; CVC, cardiovascular comorbidities; LLD, late-life depression; LLD + CVC, late-life depression with cardiovascular comorbidities. ^a^The direction of '>’ shows performance from better to worse.

The *post hoc* analysis showed that overall the HC group had the best performance and that the LLD + CVC group had the worst performance. No significant differences were found between the CVC and LLD groups or between the LLD and LLD + CVC groups on most WCST indices. However, there were significant differences between the CVC and LLD + CVC on most tested indices: the LLD + CVC group showed significantly greater cognitive dysfunction. Moreover, for performance on the TMT, because of the participants' age and the psychomotor speed requirement of the test, some participants could not complete both the TMT-A and TMT-B (29 HC, 78 CVC, 28 LLD, and 28 LLD + CVC group members completed the TMT-B); the nonparametric Kruskal-Wallis test was then used to analyze the scores. The results were similar to those on the WCST: the HC group had the best performance and the LLD + CVC group had the worst (Table 3).

**Table 3:** Comparison of executive functions between four groups of older elderly men (≥75 years old)

	**HC**	**CVC**	**LLD**	**LLD + CVC**	**ANOVA**	***Post hoc***^**a**^
	**(*n*** **= 33)**	**(*n*** **= 101)**	**(*n*** **= 34)**	**(*n*** **= 49)**	**( *F *, *p *** **value)/*χ***^**2**^	
TNE	57.58 ± 23.43	68.75 ± 19.16	74.71 ± 13.12	80.02 ± 17.59	(*F* = 10.31, *p* < 0.0005)	HC > CVC > LLD + CVC, HC > LLD^b^
PE	37.09 ± 20.22	38.21 ± 21.05	38.03 ± 17.91	47.18 ± 26.01	(*F* = 2.31, *p* = 0.08)	HC > LLD + CVC (*p* = 0.04), CVC > LLD + CVC^b^
CLR	42.00 ± 21.74	30.86 ± 18.13	27.24 ± 14.21	18.24 ± 16.29	(*F* = 11.86, *p* < 0.0005)	HC > CVC, LLD, LLD + CVC; CVC > LLD + CVC^b^
NCC	3.33 ± 1.76	1.92 ± 1.55	1.21 ± 1.01	0.71 ± 1.08	(*F* = 24.47, *p* < 0.0005)	HC > CVC, LLD + CVC, HC > LLD^b^
TCFC	34.09 ± 31.83	54.57 ± 42.99	70.82 ± 42.53	94.35 ± 46.32	(*F* = 15.87, *p* < 0.0005)	HC > LLD, LLD + CVC; CVC > LLD + CVC; HC > CVC^b^
TMT-A	122.09 ± 92.76	128.57 ± 75.85	126.26 ± 31.69	148.06 ± 73.52	(*χ*^2^ = 8.93, *p* = 0.03)^c^	HC > LLD (*p* = 0.047), LLD + CVC, CVC > LLD + CVC (*p* = 0.036)^d^
TMT-B	2462.21 ± 147.96	280.86 ± 107.84	265.82 ± 50.92	326.18 ± 91.67	(*χ*^2^ = 9.93, *p* = 0.02)^c^	HC > LLD + CVD, CVC > LLD + CVC (*p* = 0.049), LLD > LLD + CVC^d^
TMTB-A	140.28 ± 75.19	151.21 ± 72.31	136.36 ± 43.74	173.86 ± 48.76	(*χ*^2^ = 9.49, *p* = 0.02)^c^	HC > LLD + CVD, CVC > LLD + CVC (*p* = 0.044), LLD > LLD + CVC^d^
TMTB/A	2.62 ± 1.08	2.47 ± 0.94	2.11 ± 0.63	2.31 ± 0.66	(*χ*^2^ = 3.58, *p* = 0.31)^c^	-

Data are presented as means ± SD. HC, healthy controls; CVC, older elderly men with cardiovascular comorbidities; LLD, older elderly men with late-life depression; LLD + CVC, older elderly men with late-life depression with cardiovascular comorbidities; TNE, total number of errors; PE, perseverative errors; CLR, conceptual level response; NCC, number of categories completed; TCC, trials to complete the first category. ^a^The direction of '>’ shows the performance from better to worse; ^b^Fisher's least significant difference post hoc test; ^c^Kruskal-Wallis test; ^d^Mann-Whitney *U* test.

### Correlation between depression and cognitive function

Because there were either no significant differences or only borderline differences at most in neuropsychological functions between the LLD + CVC and LLD groups, these two groups were then combined as one to further investigate the effect of depressive symptoms on these cognitive functions, using GDS subscales [13]. Mood status, inferiority, and disinterest were significantly associated with neurocognitive performance, especially on the TMT tasks (Table 4).

**Table 4:** **Correlation (Spearman's *ρ*) between depressive symptoms and neuropsychological functions in patients with depression on the GDS**

	**Geriatric depression scale**			
	**Negative mood**	**Energy level**^**a**^	**Inferiority**	**Disinterest**
Mini-mental atate examination				
Orientation	-0.16 (0.16)	0.13 (0.25)	0.00 (0.99)	0.07 (0.52)
Registration	-0.05 (0.67)	-0.11 (0.33)	0.08 (0.47)	0.08 (0.49)
Attention	-0.03 (0.79)	0.04 (0.69)	0.16 (0.14)	-0.08 (0.47)
Recall memory	-0.04 (0.73)	-0.11 (0.32)	0.01 (0.92)	0.13 (0.25)
Language	-0.05 (0.63)	0.08 (0.46)	-0.05 (0.67)	-0.02 (0.89)
Spatial concept	0.01 (0.90)	-0.10 (0.39)	-0.11 (0.32)	-0.24 (0.03)
Trail Making Test A	-0.19 (0.90)	0.10 (0.36)	-0.21 (0.07)	-0.01 (0.78)
Trail Making Test B	0.11 (0.41)	-0.09 (0.50)	*-*0.28 (0.035)	-0.04 (0.79)
Trail Making Test B-A	0.28 (0.04)	-0.20 (0.15)	-0.17 (0.22)	-0.18 (0.18)
Trail Making Test B/A	0.29 (0.03)	-0.09 (0.53)	0.06 (0.66)	-0.31 (0.02)

Data are presented as *ρ* (*p* value). ^a^The higher score represents the lower energy level.

## Discussion

We found that the LLD + CVC group had the lowest recall memory scores of the four groups. This indicated that depression might affect memory more than it affects the other functions tested by the MMSE, which might be used for preliminary screening of the older elderly. Kramer-Ginsberg et al. [23] found no significant differences in the Animal Naming tests (executive domain) or in the General Memory index of the revised Wechsler Memory Scale, but a significantly lower score was found in the Delayed Recall index, which was similar to our finding. This might imply that the depressed older elderly could have moderate-to-severe deep white matter MRI signal hyperintensities, which indicate perivascular lesions caused by 'dilated perivascular spaces (with and without ischemia in the perivascular area), oligemic demyelination, [or] ischemic demyelination’ [24]. Regular follow-ups are necessary to search for additional organic and structural changes, such as Parkinson's disease, dementia, and other neurodegenerative diseases, in the brains of the depressed older elderly.

The LLD + CVC group had the lowest executive function scores of the four groups, which is consistent with previous reports [25]. There were no significant differences between the CVC and the LLD groups or the LLD and the LLD + CVC groups, but there were significant differences between the CVC and the LLD + CVC groups for most of the tested indices. This implied that LLD has a far more important effect on neurocognitive impairment than do CVCs in the older elderly.

The explanation for the nonsignificant differences between the LLD and LLD + CVC groups might be a similar pathogenesis of depression and cardiovascular comorbidities, such as neuroendocrine dysfunctions, inflammatory processes, autonomic dysregulation, platelet abnormalities, and certain vulnerability genes [26], all of which may lead to cognitive deficits [27] and to a cumulative effect of a comorbidity on the degree of cognitive deficit [28].

Although we found no significant differences between the CVC and the LLD groups in the MMSE testing, we did find significant differences between their scores on the WCST and the TMT. On the WCST, the LLD group had significantly lower scores than did the CVC group, but on the TMT, the CVC group took a significantly longer time to complete the task. This suggests that LLD and CVC have different neurocognitive effects in the older elderly. Although both tools are used to examine executive function in general, some differences between them should be taken into account. The TMT tests psychomotor performance and the WCST tests reasoning. Planning might be more affected by cardiovascular comorbidities than by depression. Moreover, the CVC group might have mild but asymptomatic cardiovascular comorbidities such as atrial fibrillation, which could affect executive function and psychomotor tasks. Even in the absence of manifest stroke, atrial fibrillation is a risk factor for memory impairment and hippocampal atrophy [29,30].

In the older elderly, negative moods, feelings of inferiority, and disinterest seem to have an important effect on neurocognitive performance, one that is associated primarily with psychomotor retardation rather than with other cognitive functions. That the LLD group patients in this study were much slower than the CVC group patients was probably because of their depression [31]. In addition, looking into the questions of this subscale, all questions are about satisfaction with the quality of the respondent's quality of life (QoL) (satisfied with my current life, often feel bored, often feel lonely, often afraid unlucky things will happen, feel unhappy most of the time, and feel unhappy being alive). This correlation might reflect the evidence that the older elderly group who live without relatives would experience greater impact on their cognitive performance, especially those with depression. No association between LLD and GDS scores on the energy level was found, possibly because both aging and depression lower their energy and this index of GDS may not be as sensitive in distinguishing the effect between aging and depression in the older elderly. Although a negative correlation was found between TMT (B/A) and GDS in the disinterest level, this might reflect a curvilinear correlation with cerebral impairment, as Corrigan and Hinkeldey [32] suggested. They also reported that TMT (B/A) was more sensitive than TMT (B-A) in differentiating lateralized damage but may not relate to impairment. The negative association between the inferiority subitem and the TMT-B score might reflect that these veterans may need more attention from more different people, not just the caregivers they see every day in the hospital, but less attention related to their actual cognitive impairment. In addition, the patients' energy levels could be affected by their QoL [33]; moreover, the onset and duration of depression might have another effect and requires further study.

The findings of this study may give psychiatric clinicians and primary care physicians an indication that mood and self-esteem should be taken into account while caring for the older elderly, whose memories are more vulnerable than those of the younger elderly to the adverse effects of negative emotional states [34]. In addition, most of the older elderly recruited in this study lacked family support and lived alone, which may have lowered their self-esteem and increased their negative emotions. Providing access to primary care institutions and other caregivers in a community-based program might be helpful for this older elderly group.

The findings of the present study might not be generalizable to other cohorts of older elderly because all of our participants were men. The older elderly should also be studied to see whether there are significant gender-based differences. Moreover, the small sample size in both the LLD and LLD + CVC groups might limit the applicability of the results for the effect of depression on memory in the older elderly. Using the MMSE as a screening instrument to make a diagnosis might also be a limitation. In addition, the variability of the cardiovascular comorbidities in the CVC group could affect different cognitive domains and limit the applicability of our conclusion. Further focusing on specific diseases is needed to clarify the effect of depression on the older elderly.

## Competing interests

The authors declare that they have no competing interests.

## Authors' contributions

YHC wrote the first draft of this manuscript under the supervision of RBL. MEL, CCH, SYL, SLC, YCK, and WCL managed the patients' recruitment and psychological testing with YHC. All authors read and approved the final manuscript.
